# Sulfakinin Signaling Sense Circulating Fructose and Suppresses Food Consumption via Insulin‐Like Peptide in *Bactrocera Dorsalis*

**DOI:** 10.1002/advs.202514748

**Published:** 2026-03-12

**Authors:** Hong‐Fei Li, Bao Dong, Zheng‐Lin Ren, Xue‐Guang Zhu, Quan Lei, Yoonseong Park, Jin‐Jun Wang, Hong‐Bo Jiang

**Affiliations:** ^1^ Key Laboratory of Entomology and Pest Control Engineering College of Plant Protection Key Laboratory of Agricultural Biosafety and Green Production of Upper Yangtze River (Ministry of Education) Academy of Agricultural Sciences Southwest University Chongqing China; ^2^ Department of Entomology Kansas State University Manhattan Kansas USA

**Keywords:** feeding, fructose, insulin‐like peptide, nutrient sensing, sulfakinin

## Abstract

Feeding in animals is primarily regulated by the internal satiety state. Sulfakinin (Sk) is well known as a satiety signal that inhibits feeding. However, the underlying mechanism activating the Sk release is not yet fully understood, although the Sk for inhibitions of peripheral sensory systems, gustatory sweet and olfactory food smell sensors, were previously described. Here, in further investigations of Sk signaling pathways, we found the upstream and an additional downstream mechanism by using CRISPR/Cas9‐mediated gene knockouts for the upstream gustatory receptor, *Gr43a*, and the downstream sulfakinin receptor 1, *SkR1*, in the oriental fruit fly. We demonstrated that the increased hemolymph fructose is sensed by GR43a, which activates the Sk signal in the intercerebralis insuline‐like peptide (Sk‐ILP) cells. The Sk‐ILP acts as an autocrine signal activating the insulin‐like peptide 5 (*ILP5*) as the ultimate satiety signal. This study demonstrated a fructose‐Gr43a‐Sk‐SkR1‐ILP5 pathway that regulates feeding behavior by sensing nutritional state.

## Graphical abstract

This study discovered a new pathway that tells fruit flies when to stop eating. It found that rising blood sugar (fructose) is detected by a sensor called GR43a. This triggers a chain reaction involving the satiety signal sulfakinin and its receptor, ultimately activating a final satiety signal, ILP5. This pathway directly links nutrient levels to feeding behavior.

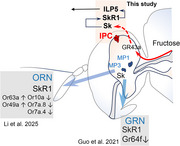

## 1 Introduction

Sensing satiety is essential to ensure a balanced intake of essential nutrients. In rodent models, several groups of hypothalamic neurons have been identified as satiety sensors [1]. These neurons sense organismal satiety through multiple pathways, including nutrients in the circulatory system, gastrointestinal satiety, and fat depots, and inhibit food consumption accordingly [2]. In *Drosophila melanogaster*, insulin‐producing cells (IPCs) in the brain are important in the regulation of feeding and metabolism. IPCs can control appropriate feeding by sensing nutrients directly in the hemolymph or indirectly through upstream neuronal populations [3, 4, 5, 6].

Hemolymph circulating sugars, including glucose, trehalose, and fructose, are all important nutrients used by IPCs to assess nutritional states in *D. melanogaster*. The sugar‐sensing mechanisms in IPCs are through upstream neuronal populations to indirectly sense nutritional states [7, 8]. For example, adipokinetic hormone (AKH)‐producing cells in the corpora cardiaca are able to sense glucose and activate IPCs to release sugar‐dependent dilp3 via AKH signaling [9]. Consistent with this, two pairs of DTK neurons located in the superior medial protocerebrum region of the *Drosophila* brain can directly sense D‐glucose in the hemolymph and inhibit overfeeding by targeting IPCs via TAKR99D neurons [4]. *D. melanogaster* also senses glucose levels via a pair of glucose‐sensing neurons in the brain expressing short neuropeptide F (sNPF) and corazonin (Crz), and fructose via Gr43a‐expressing neurons in the brain [5, 10]. In addition, IPCs can detect other nutrients besides hemolymph sugars. For example, IPCs can directly sense extracellular leucine levels via minidiscs [6]. It is not yet fully uncovered how numerous feeding modulatory cues work in concert with IPCs for appropriate regulation of feeding behavior, while there are numerous signals controlling the feeding, including NPF, Hugin, drosulfakinin (DSK), and leukokinin (LK) [11].

Sulfakinin (Sk) is a neuropeptide known to inhibit feeding behavior, homologous to the mammalian peptide cholecystokinin (CCK) [12]. In *D. melanogaster*, feeding increases the expression of sulfakinin, as well as the activity of a group of Sk neurons [13]. Sk reduces the expression of the gustatory receptor *Gr64f* by activating the receptor CCKLR‐17D3 neurons located in the proboscis and proleg tarsi [13, 14]. Then, feeding is suppressed due to reduced activity of sweet‐sensing gustatory neurons mediated by *Gr64f*. In addition to the gustatory system, Sk also regulates feeding behavior through the olfactory system. In *Bactrocera dorsalis*, Sk enhances foraging behavior by altering the olfactory representation of starved flies [15]. However, it has been shown that Sk signaling can inhibit feeding in a number of ways in addition to the peripheral system [12]. In *Locusta migratoria*, SKs can inhibit the activity of digestive enzymes to regulate food consumption [16]. Similarly, CCK stimulates pancreatic enzyme secretion and the release of insulin and glucagon in mammals [17]. Thus, it remains unknown whether Sk regulates insect feeding behavior at the level of internal nutritional state.

Here, we found that Sk also suppresses feeding by sensing internal nutrients, in addition to inhibiting peripheral taste sensitivity. We found sweet caloric sugar diet causes a significant upregulation of fructose in the hemolymph of *B. dorsalis*. Gr43a neurons sense fructose and convey nutrient signals to Sk neurons in the pars intercerebralis (PI) region, including IPC, ultimately inducing Sk release. Sk transmits satiety signals to IPCs by activating SkR1. Additionally, the Sk‐SkR1 signal may directly act on IPCs to regulate feeding behavior. Collectively, our study illuminates a fructose‐Gr43a‐Sk‐SkR1‐ILP5 pathway that modulates feeding behavior by sensing nutritional state.

## 2 Results

### 2.1 Satiety Induces Sk Release That Inhibits Feeding

**FIGURE 1:**
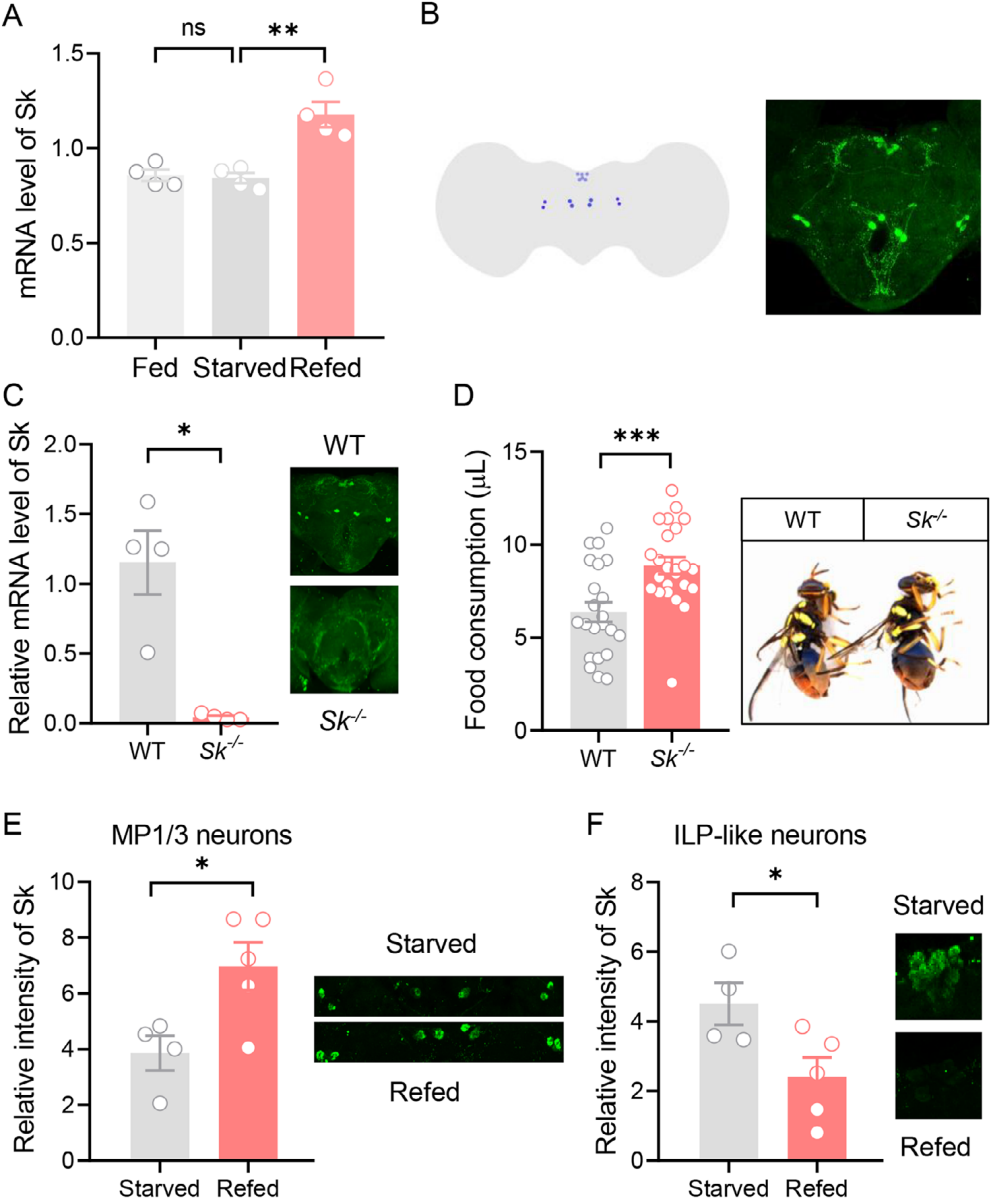
Feeding induces Sk release, and Sk inhibits feeding in *B. dorsalis*. (A) Sk mRNA level was significantly increased in the refed brain. *n* = 4. (B) Expression pattern of Sk neurons in the brain. Two groups of Sk neurons were localized in the brain. One group was a symmetrically distributed four pairs of neurons located in the medial protocerebrum (MP) region, named Sk‐MP neurons. The other group was a cluster‐distributed neuron located in the pars intercerebralis (PI) region, with a similar location to insulin neurons, named Sk‐ILP neurons. (C) CRISPR‐Cas9‐directed mutagenesis of Sk eliminated the expression of Sk. *Sk^−/−^* flies have no Sk mRNA expression in the brain by qRT‐PCR analysis. *n* = 4. The right panel showed that *Sk^−/−^* brain showed no anti‐Sk positive signal by immunolabeling. (D) Knockout of Sk increased the food consumption of flies. *n* = 17 and 25 flies, respectively. The right panel showed representative images of WT and *Sk^−/−^* flies. (E) The intensity of immunoreactivity in Sk‐MP neurons of starved and refed flies. The intensity of immunoreactivity in Sk‐MP neurons of starved flies was significantly lower than that in Sk‐MP neurons of refed flies. The right panel showed representative images of Sk‐MP neurons. (F) The intensity of immunoreactivity in Sk‐ILP neurons of starved and refed flies. The intensity of immunoreactivity in Sk‐ILP neurons of starved flies was significantly higher than that in Sk‐ILP neurons of refed flies. The right panel showed representative images of Sk‐ILP neurons. *n* = 4 and 5 flies, respectively. All data are plotted as mean ± SEM. Unpaired *t* test, ^*^ *p* < 0.05, ^**^ *p* < 0.01, ^***^ *p* < 0.001.

It has been shown that Sk mediates satiety‐induced suppression of feeding [13]. Consistently, Sk signaling was also highly expressed in the *B. dorsalis* brain (Figure S1). We first examined the response of Sk signaling to the feeding state. We examined the mRNA levels of Sk by qPCR and in situ hybridization in the flies that had been starved for 12 h, and then the starvation was followed by 1 h of feeding. The results showed that the expression of Sk was significantly higher in refed flies than in fed and starved flies (Figure 1A). Notably, we localized two groups of Sk neurons in the brain. One group was a symmetrically distributed four pairs of neurons located in the medial protocerebrum (MP), named Sk‐MP neurons. The other group was a cluster‐distributed neuron located in the pars intercerebralis (PI) region, with a similar location to insulin‐like peptide cells, named Sk‐ILP neurons (Figure 1B). Sk mRNA levels by in situ hybridization were significantly higher after refeeding both in Sk‐ILP and Sk‐MP neurons (Figure S2). Subsequently, Sk null mutant, *Sk^−/−^*, was generated using CRISPR/Cas9 system [15]. We further validated the *Sk^−/−^* mutant at the mRNA and peptide levels. As expected, no Sk mRNA was found, and no anti‐Sk immunopositive signal was found in the *Sk^−/−^* brains (Figure 1C). Moreover, the immunoreactivity of SK‐MP and Sk‐ILP neurons in the *Sk^−/−^* mutants all disappeared, suggesting that these two groups of neurons do express Sk (Figure 1C). We also measured the food consumption of *B. dorsalis* using dye‐labeled food (Figure S3). The results showed that the knockout of Sk significantly increased food consumption (Figure 1D). In summary, Sk responds to the feeding state and acts as an inhibitor of feeding.

Next, we wanted to test whether the feeding state not only regulates Sk mRNA expression but also plays a role in the release of Sk peptides. Brains from starved and refed flies were dissected to reveal the amount of Sk stored in Sk neurons by anti‐Sk staining. The results showed that the intensity of immunoreactivity in Sk‐MP neurons of starved flies was significantly lower than that in Sk‐MP neurons of refed flies (Figure 1E). In contrast, the intensity of immunoreactivity in Sk‐ILP neurons of starved flies was significantly higher than that in Sk‐ILP neurons of refed flies (Figure 1F), suggesting that feeding induces Sk release in Sk‐ILP neurons. We also performed a time‐course experiment, measuring Sk immunoreactivities at 15 min, 30 min, 1 h, and 2 h post‐feeding. Results showed that Sk‐MP neurons showed higher intensity at 1 and 2 h post‐feeding, but no significant changes at 15 and 30 min post‐feeding (Figure S4A). Sk‐ILP neurons exhibited lower intensity at 1 h post‐feeding, higher intensity at 2 h post‐feeding, while no significant changes occurred at 15 and 30 min post‐feeding (Figure S4B). Therefore, we conclude that the satiety signal activates *Sk* transcript in both Sk‐MP and Sk‐ILP cells, while it also triggers the Sk release mainly in Sk‐ILP cells at 1 h post‐feeding.

### 2.2 Sweet and Caloric Sugars Induce Sk Release

**FIGURE 2:**
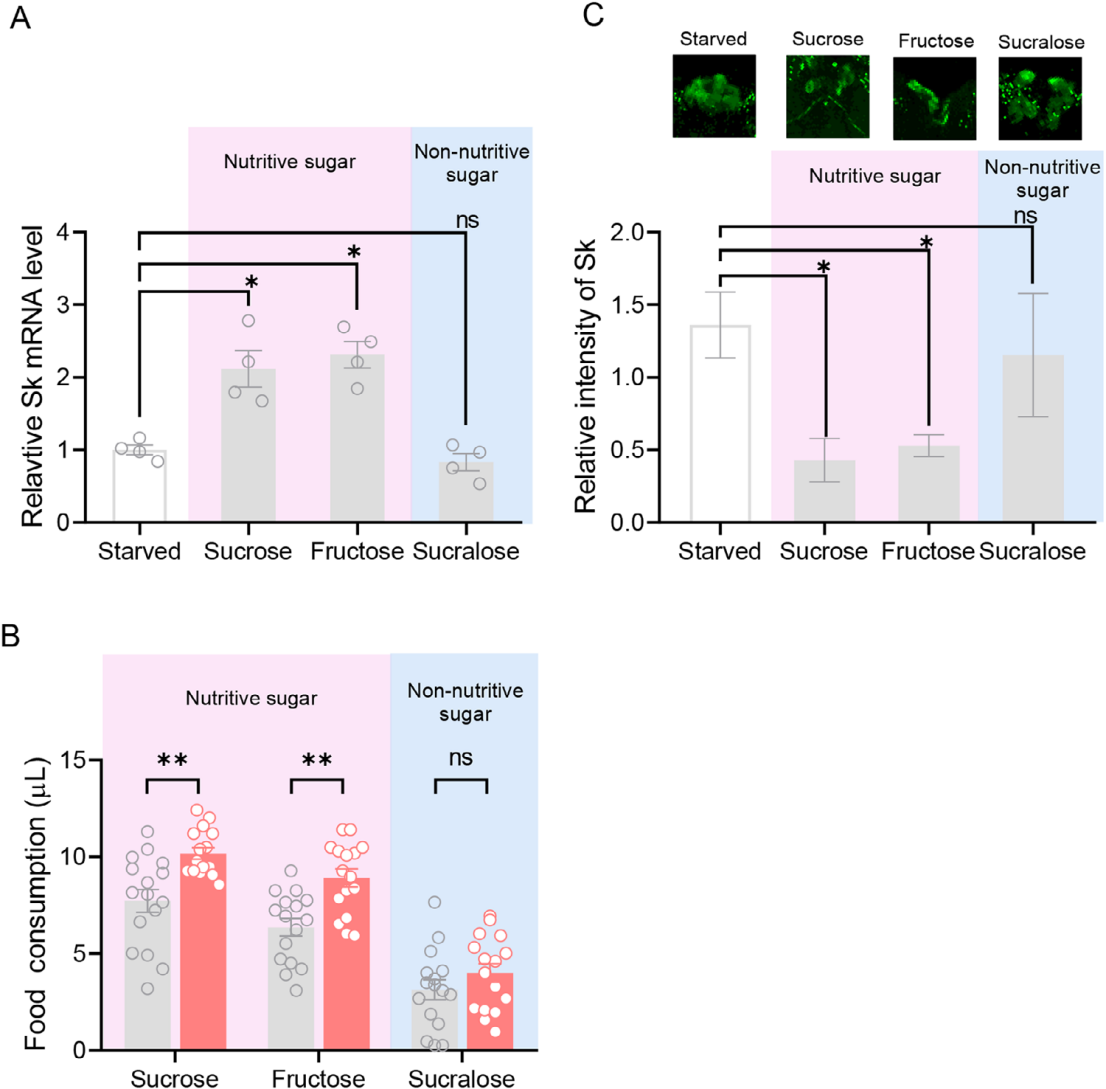
Sk responds to sucrose and fructose. (A) Changes in Sk mRNA levels of flies fed different sugars. Expression of Sk was significantly increased after feeding sucrose and fructose. *n* = 4. (B) Changes in consumption of different sugars by *Sk^−/−^* flies. Food consumption of sucrose or fructose was significantly increased in *Sk^−/−^* flies. Each experiment consisted of one fly, and at least 16 flies were contained for the assay. (C) Quantification of anti‐Sk signal in Sk‐ILP neurons of flies fed different sugars. The intensity of immunoreactivity in Sk‐ILP neurons of starved flies was significantly stronger than that in Sk‐ILP neurons of flies fed sucrose or fructose. The top panel showed representative images of Sk‐ILP neurons. *n* = 4–5. All data are plotted as mean ± SEM. Unpaired *t*‐test, ns indicates no significant difference, ^*^ *p* < 0.05, ^**^ *p* < 0.01.

Since Sk can inhibit feeding by reducing the sweetness sensitivity to sucrose, we sought to identify whether sweet taste is sufficient in Sk‐mediated feeding inhibition [13]. Sucrose is a dimer of fructose and glucose, and glucose is sensed by tachykinin neurons. Therefore, we selected three types of sugars to feed flies, including sucrose (sweet and caloric), fructose (sweet and caloric), and sucralose (sweet but not caloric). We found that *Sk* mRNA levels of flies fed sucralose did not change significantly compared to starved flies, while *Sk* mRNA levels of flies fed sucrose or fructose were all significantly up‐regulated (Figure 2A). We further determined the changes in feeding on different sugars in *Sk^−/−^* mutants. The results showed that *Sk^−/−^* mutants showed a higher food consumption of sucrose and fructose compared to WT; however, there was no significant change in food consumption of sucralose (Figure 2B). This is consistent with changes in Sk mRNA levels induced by different sugars. This suggests that Sk responds to sweet caloric sugars, not only in relation to sweetness.

To further validate this observation, we investigated the amount of Sk stored in Sk‐ILP neurons of flies after feeding different sugars by anti‐Sk staining. The results showed that the intensity of Sk immunoreactivity in Sk‐ILP neurons of starved flies was significantly stronger than that in Sk‐ILP neurons of flies fed sucrose and fructose (Figure 2C); however, there was no significant change in the intensity of immunoreactivity in Sk‐ILP neurons of flies fed sucralose compared with starved flies (Figure 2C). This is consistent with the feeding phenotype, which suggests that it is sweet caloric sugar that induces SK release in Sk‐ILP neurons.

### 2.3 Fructose From Dietary Fructose or Nutritive Sugar Metabolism Induces Sk Release

**FIGURE 3:**
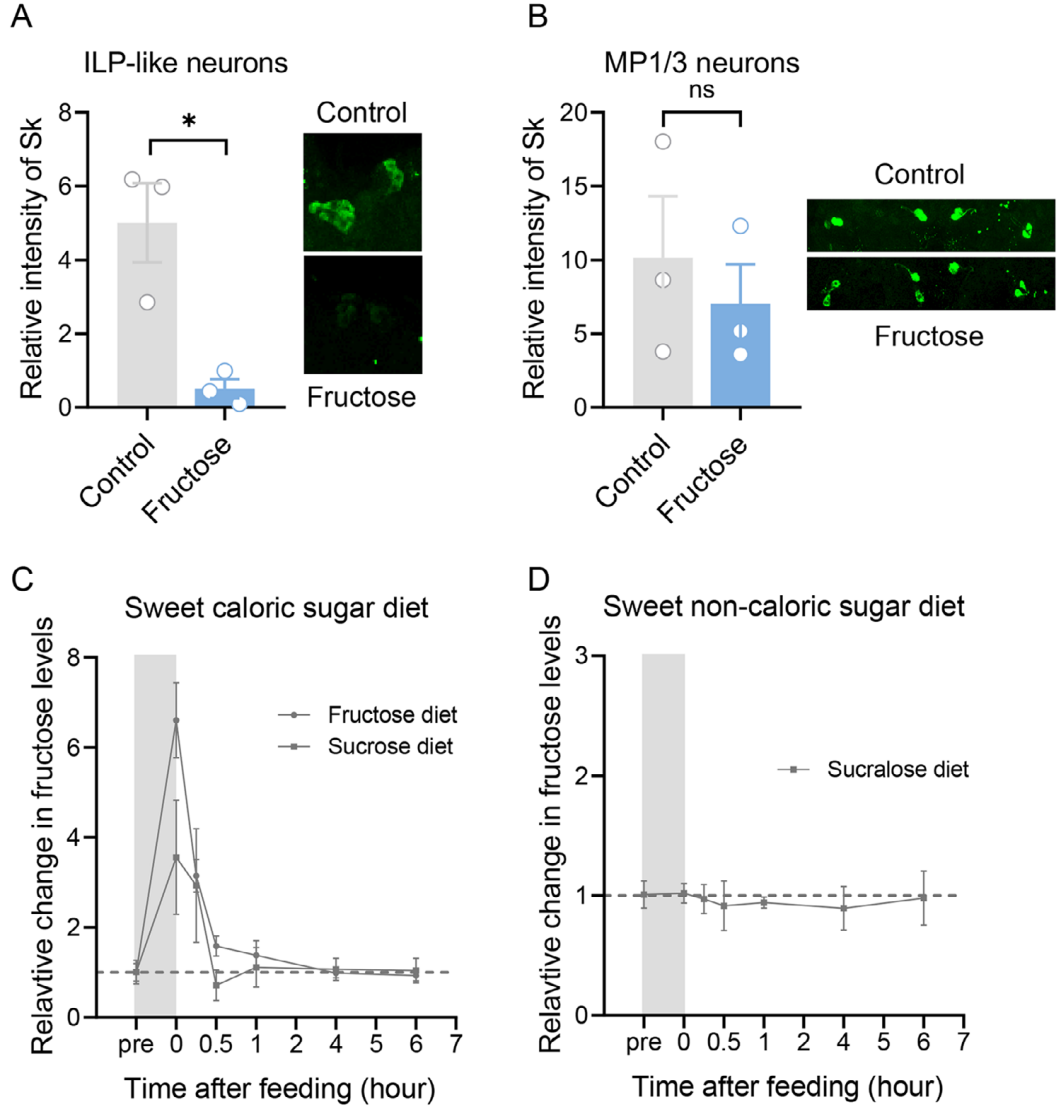
Fructose induces Sk release. Flies were starved for 24 h (pre), followed by 30 min of feeding. Measurements were performed at the indicated times after feeding, and adult male heads were used for measurement. (A) Immunoreactivity of intracellular Sk in Sk‐ILP neurons when the brains were incubated in 80 mm fructose in an artificial haemolymph‐like solution (AHL). *n* = 3. The right panel showed representative images of Sk‐ILP neurons. (B) Immunoreactivity of intracellular Sk in Sk‐MP neurons when the brains were incubated in 80 mm fructose in AHL. *n* = 3. The right panel showed representative images of Sk‐MP neurons. Data are plotted as mean ± SEM. Unpaired *t*‐test, ns indicates no significant difference, ^*^ *p* < 0.05. (C) Relative change of internal fructose over time after a sweet caloric sugar diet. (D) Relative change of internal fructose over time after a sweet non‐caloric sugar diet. *n* = 4. Data are plotted as mean ± SEM.

In flies, fructose is one of three main hemolymph sugars and serves as a signal for an internal nutrient sensor [10]. Therefore, we further clarified whether fructose induces Sk release. Brains were exposed to adult hemolymph‐like solutions (AHL) mixed with the final concentration of 80 mm fructose, and anti‐Sk staining revealed the amount of Sk storage in *B. dorsalis* Sk‐ILP neurons after incubation. The results showed that fructose incubation significantly reduced the intensity of immunoreactivity in Sk‐ILP neurons but not in Sk‐MP neurons (Figure 3A, B). This suggests that fructose induces Sk release in Sk‐ILP neurons. Next, we determined whether the ingestion of sucrose or fructose altered the levels of circulating fructose in the *B. dorsalis* brain. When flies were provided with a sucrose or fructose meal, fructose levels sharply increased by 3.6 and 6.6 fold, respectively (Figure 3B). However, feeding sucralose failed to affect fructose levels (Figure 3C). The relative change in circulating fructose dynamics after different sugar diets further supports that fructose induces Sk release.

### 2.4 Fructose Requires Gr43a to Induce Sk Release to Inhibit Feeding

**FIGURE 4:**
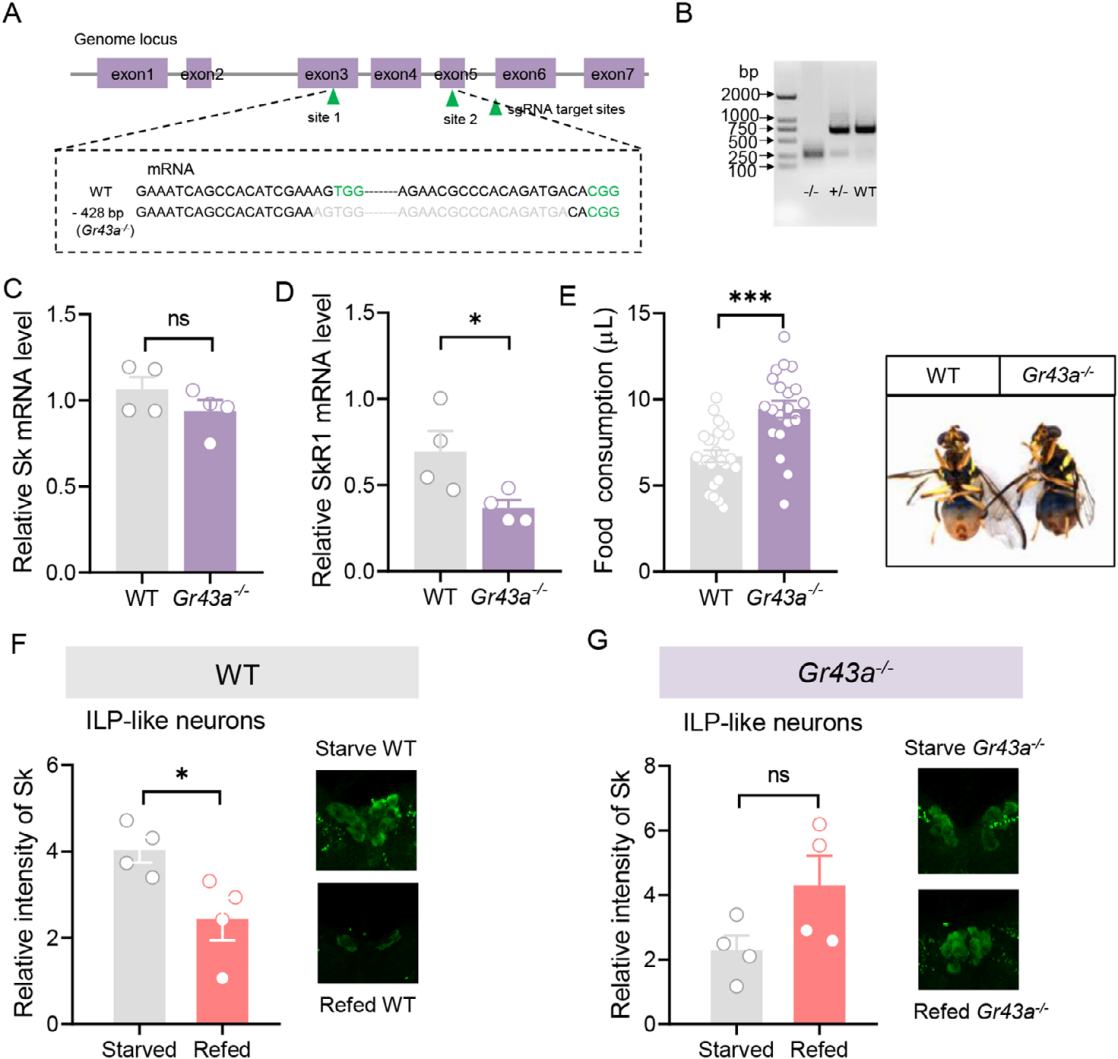
Knockout of *Gr43a* promotes feeding in *B. dorsalis*. (A) Schematic of the *Gr43a* gene, showing the two guide RNA (sgRNA) target sites. The *Gr43a* gene has seven exons, shown as purple boxes. A 428‐bp deletion was chosen for the generation of *Gr43a^−/−^* flies. The protospacer adjacent motif (PAM) is highlighted in green. (B) Gel electropherogram of *Gr43a* from WT, *Gr43a^+/−^* and *Gr43a^−/−^*. (C) Knockout of *Gr43a* has no impact on the expression of the Sk gene in flies. *n* = 4. (D) Knockout of *Gr43a* reduced expression of *SkR1* gene in flies. *n* = 4. (E) Knockout of *Gr43a* increased food consumption of flies. The right panel represents representative images of WT and *Gr43a^−/−^* flies that ingested food with dye. *n* = 24 and 21 flies, respectively. (F) Quantification of anti‐Sk signal in Sk‐ILP neurons of starved WT and refed WT flies. The intensity of immunoreactivity in Sk‐ILP neurons of starved WT flies was significantly stronger than that in Sk‐ILP neurons of refed WT flies. The right panel showed representative images of Sk‐ILP neurons. *n* = 4. (G) Quantification of anti‐Sk signal in Sk‐ILP neurons of starved *Gr43a^−/−^* and refed *Gr43a^−/−^* flies. The intensity of immunoreactivity in Sk‐ILP neurons of starved *Gr43a^−/−^* flies was the same as that in Sk‐ILP neurons of refed *Gr43a^−/−^* flies. The right panel showed representative images of Sk‐ILP neurons. *n* = 4. All data are plotted as mean ± SEM. Unpaired *t*‐test, ns indicates no significant difference, ^*^ *p* < 0.05, ^***^ *p* < 0.001.

In the fly brain, *Gr43a* encodes a narrowly tuned fructose receptor [10]. We then asked whether fructose induces Sk release via Gr43a. CRISPR/Cas9 technology was used to generate a *Gr43a* null mutant with a 428‐bp deletion (Figure 4A). The gel electrophoresis further confirmed a large deletion of the *Gr43a* gene fragments in the genome (Figure 4B). We found that knockout of *Gr43a* did not significantly change the mRNA level of *Sk* (Figure 4C), but resulted in significant downregulation of its receptor *SKR1* (Figure 4D). Consistent with this, *Gr43a^−/−^* mutant showed a higher food consumption than WT (Figure 4E and Figure S5). Immunohistochemical results showed that the fluorescence intensity of Sk‐ILP neurons was significantly stronger in WT refed flies than in WT starved flies (Figure 4F). However, in *Gr43a^−/−^* mutant flies, the fluorescence intensity of Sk‐ILP neurons in starved flies was the same as that in refed flies (Figure 4G). This suggests that fructose requires Gr43a to induce Sk release in the Sk‐ILP cells.

### 2.5 Sk Inhibits Feeding by Acting on SkR1

Based on *Drosophila melanogaster* CCKLR‐17D3 (SkR1) and CCKLR‐17D1 (SkR2), SkR1 and SkR2 were predicted from the genome of *B. dorsalis*. Phylogenetic analysis clearly showed that SkR1 and SkR2 are grouped in the insect SkR groups and cluster with *Drosophila melanogaster* CCKLR‐17D3 and CCKLR‐17D1, respectively (Figure S6A). Only SkR1 has been reported to be associated with feeding among the two predicted Sk receptor genes, SkR1 and SkR2 [13]. Therefore, the accuracy of the SkR1 sequence was further validated by cDNA cloning. SkR1 is a typical GPCR with seven transmembrane structural domains encoding 713 amino acids (Figure S6B). To confirm that the SkR1 is functional, a calcium reporter assay was performed with two mature Sk peptides from *B. dorsalis*. The results showed that SkR1 was activated by sulfated Sk‐1 in a dose‐dependent manner (Figure S6C) with the effective concentration of 50% value (EC_50_) 0.95 µm for Sk‐1.

**FIGURE 5:**
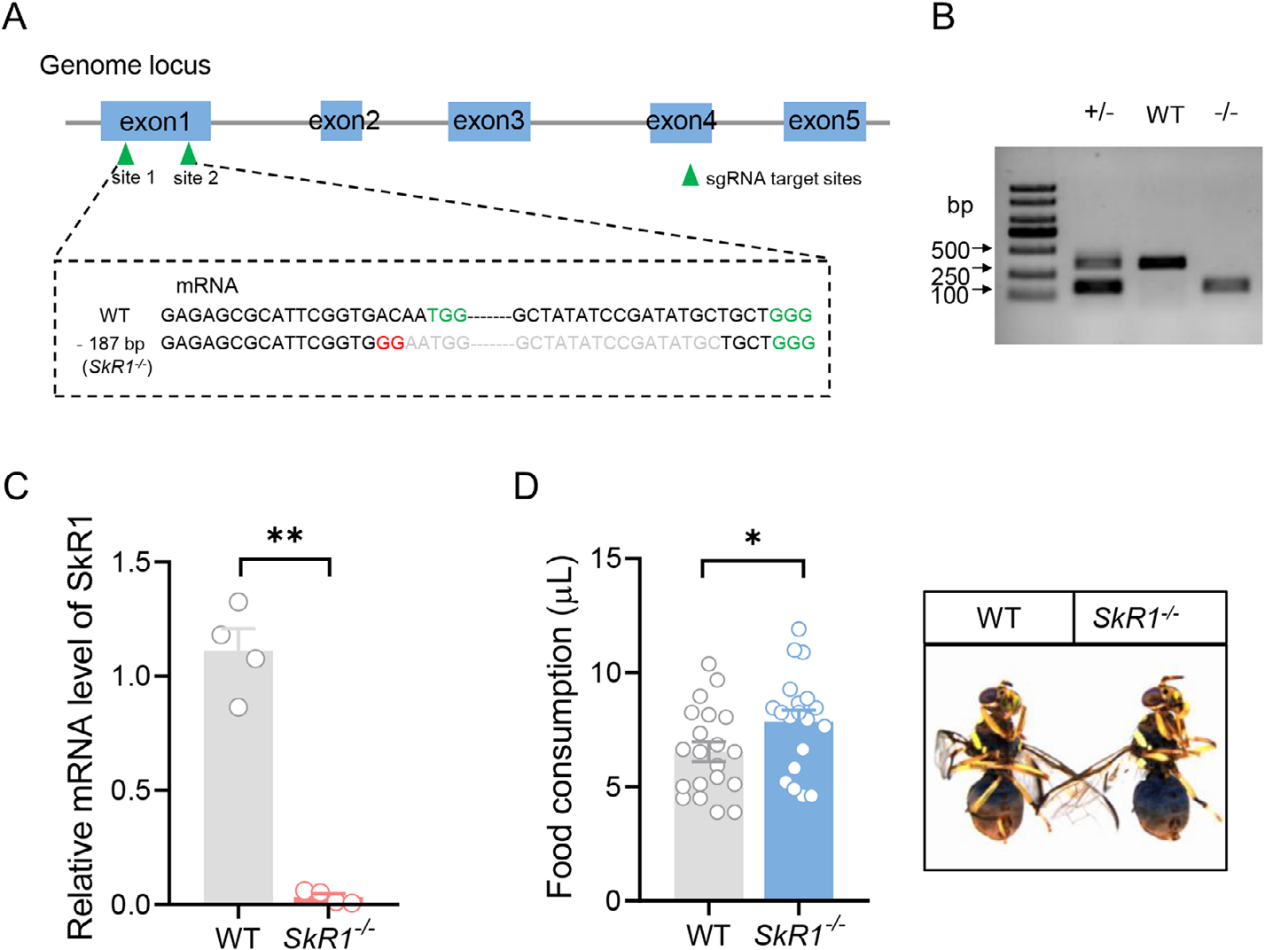
Knockout of SkR1 promotes feeding in *B. dorsalis*. (A) CRISPR/Cas9‐directed mutagenesis of *B. dorsalis SkR1*. The *BdSkR1* gene has five exons shown as blue boxes. Two sgRNA target sites were shown as arrows. A 187‐bp deletion and 2‐bp substitution were chosen for the generation of *SkR1^−/−^* flies. The protospacer adjacent motif (PAM) is highlighted in green. (B) Gel electropherogram of *SkR1* from WT, *SkR1^+/−^* and *SkR1^−/−^*. (C) Suppression of SkR1 expression in *SkR1^−/−^* flies. qPCR data showed that mRNA level of SkR1 was significantly reduced in *SkR1^−/−^* flies. *n* = 4. (D) Food consumption of *SkR1^−/−^* flies was significantly increased. The right panel represents representative images of WT and *SkR1^−/−^* flies that ingested food with dye. *n* = 20 and 19 flies, respectively. All data are plotted as mean ± SEM, Unpaired *t*‐test; ^*^ *p* < 0.05, ^**^ *p* < 0.01.

To further clarify whether SkR1 is involved in regulating the food consumption of *B. dorsalis*, we therefore generated a SkR1 null mutant (*SkR1^−/−^*) by introducing a 187‐bp deletion using CRISPR/Cas9 (Figure 5A, B). Compared with WT, the mRNA expression of the *SkR1^−/−^* mutant was almost zero, further confirming that the mutant is effective (Figure 5C). Consistently, in the *SkR1^−/−^* mutant, the fluorescence intensity of Sk‐ILP neurons in starved flies was the same as that in refed flies, suggesting accumulation of Sk in Sk‐ILP neurons due to impaired signaling (Figure S7). We then measured the food consumption of the *SkR1^−/−^* mutant. The results showed that the knockout of SkR1 significantly increased the food consumption of *B. dorsalis* (Figure 5C). Altogether, these results suggest that Sk suppresses feeding by acting on SkR1.

### 2.6 Sk Signaling Suppresses the Expression of *ILP5* to Inhibit Feeding in *B. Dorsalis*

To unravel the molecular mechanisms by which Sk‐SkR1 signaling regulates feeding behavior, transcriptomic libraries of starved WT and *Sk^−/−^* brains were constructed. After removing low‐quality regions, adapters, and possible contamination, we obtained 48528780 and 46100249 clean reads on average from WT brains and *Sk^−/−^* brains, respectively (Table S2). Among the clean bases we obtained, the Q20 percentage was more than 97%, and the Q30 percentage was more than 92% (Table S3). Tables S2 and S3 provide detailed information regarding library sequencing.

**FIGURE 6:**
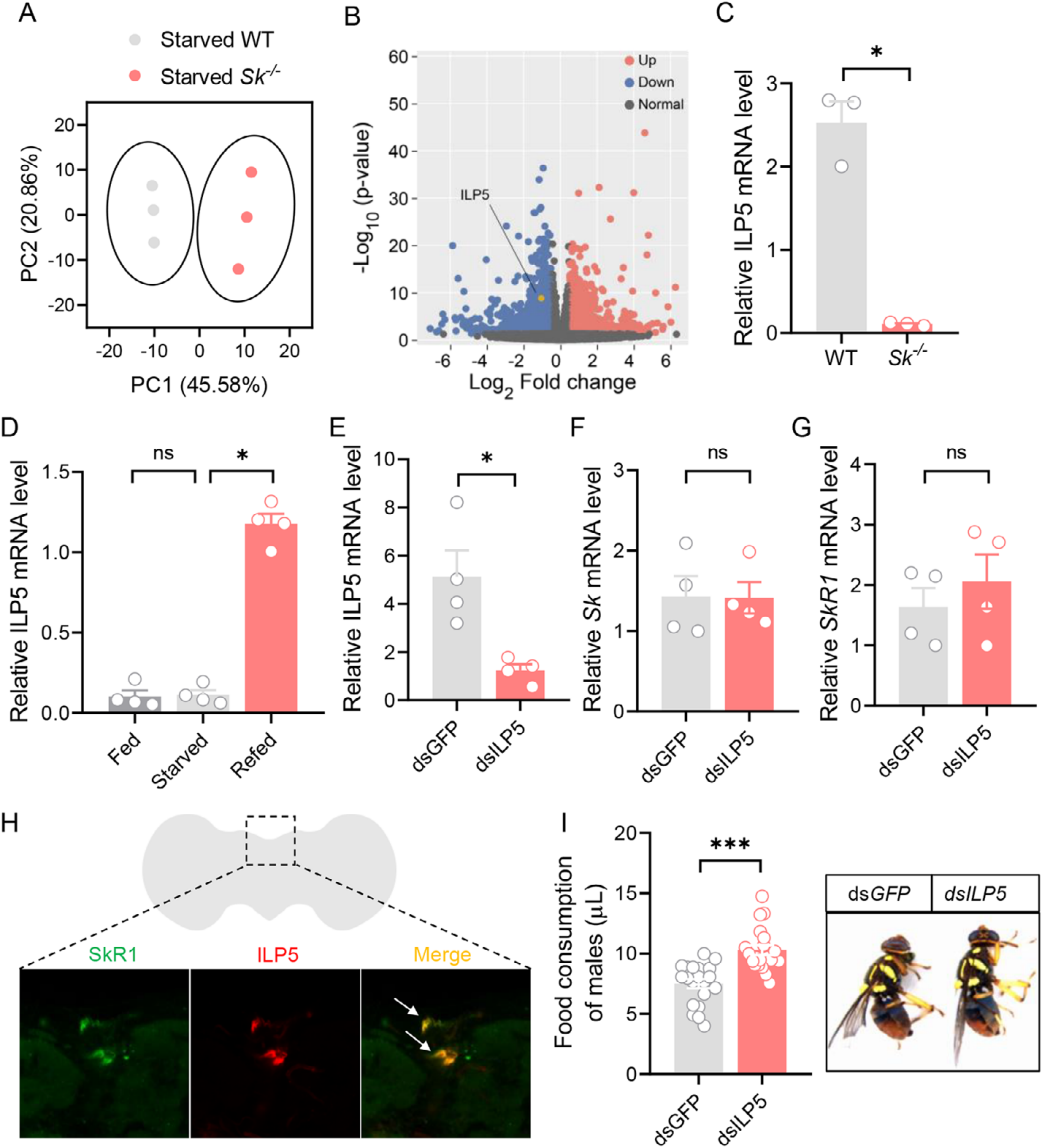
Knockout of *Sk* reduced *ILP5* expression to promote food consumption in *B. dorsalis*. (A) Principal component analysis (PCA) using differential expression genes obtained from comparisons between WT and *Sk^−/−^* brains. (B) Volcano plot of differentially expressed genes comparing WT and *Sk^−/−^* brains. Upregulated genes (*p* < 0.05 and > 2 fold) were colored red. Downregulated genes (*p* < 0.05 and > 2 fold) were colored blue. (C) RT‐qPCR result showed that knockout of *Sk* reduced the mRNA level of *ILP5* in the brains. *n* = 3. (D) Expression of ILP5 was significantly upregulated after refeeding. *n* = 4. (E) Expression of *ILP5* was significantly reduced using after dsRNA was injected. *n* = 4. (F) Knockdown of *ILP5* using RNAi had no impact on expression of the *Sk* gene in *B. dorsalis*. *n* = 4. (G) Knockdown of *ILP5* using RNAi had no impacts on expression of *SkR1* gene in *B. dorsalis*. *n* = 4. (H) SkR1 (green) and ILP5 (red) are expressed together in the PI region of the brain, as confirmed by double fluorescence in situ hybridization. (I) Knockdown of *ILP5* significantly increased the food consumption of *B. dorsalis*. The right panel represents representative images of WT and ds*ILP5*‐injected flies that ingested food with dye. *n* = 20 flies. All data are plotted as mean ± SEM. Unpaired *t*‐test, ns indicates no significant difference, ^*^ *p* < 0.05, ^***^ *p* < 0.001.

**FIGURE 7:**
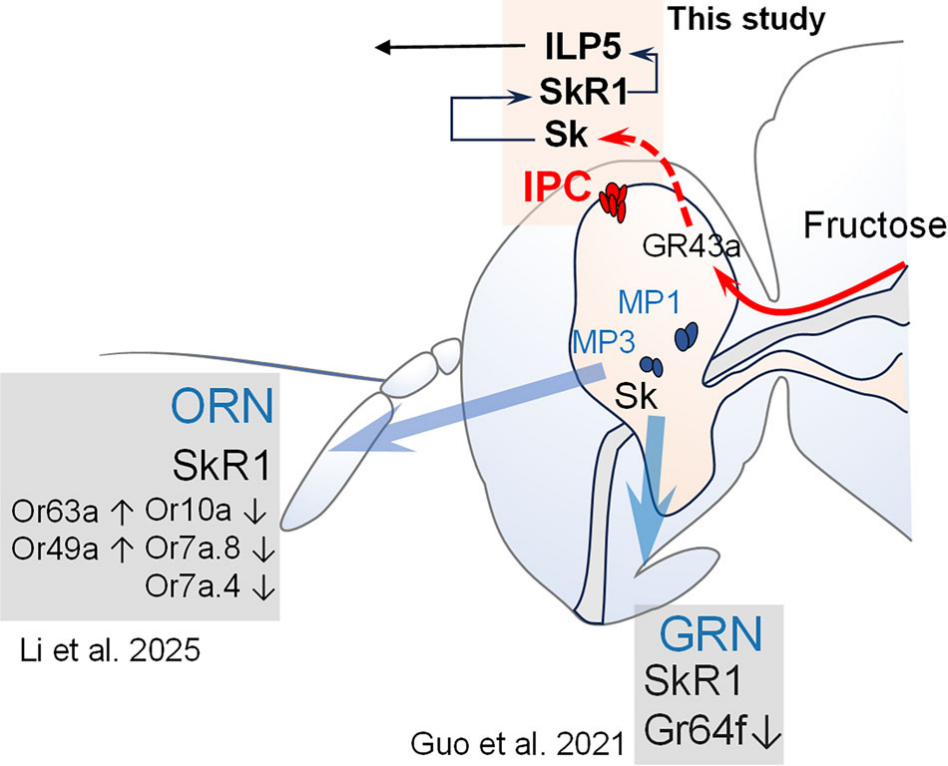
A working model of Sk as a satiety signal that coordinately inhibits feeding via parallel neuromodulatory circuits. When *B. dorsalis* feeds on nutritive sugars, fructose in the hemolymph rises significantly and is sensed by the fructose receptor Gr43a in the brain. As a result, Sk is released from Sk‐ILP neurons to activate downstream SkR1 neurons in the PI region, which subsequently elicits upregulation of *ILP5* in IPCs. Upregulation of *ILP5* inhibits feeding behaviors. In the peripheral gustatory system, when *B. dorsalis* feeds on sweet sugars, Sk is released from Sk‐MP neurons to activate downstream SkR1 neurons in the proboscis, and subsequently elicits upregulation of *takeout* in GRNs, which suppresses *Gr64f* expression. Downregulation of *Gr64f* leads to decreased sugar sensitivity and food ingestion.

Based on the FPKM values, the differentially expressed genes between the WT and *Sk^−/−^* groups were calculated. Results showed that the expression levels of 461 genes were up‐regulated in the *Sk^−/−^* group compared with the WT group; meanwhile, the expression levels of 351 genes were down‐regulated (Figure 6A, B). Gene Ontology (GO) analysis revealed an enrichment of GO terms related to translation, peptide biosynthetic process, peptide metabolic process, and hormone activity, suggesting that Sk may be involved in the synthesis or activity of some hormones (Figure S8). Consistently, we identified a neuropeptide related to feeding behavior, the insulin‐like peptide 5 (ILP5), in DEGs. Subsequently, we verified the expression changes of *ILP5* using qPCR. Consistent with the transcriptomic results, the expression of *ILP5* was significantly reduced in the *Sk^−/−^* brains (Figure 6C). In addition, we found that refeeding significantly induced *ILP5* expression in WT flies, while no changes were observed in *SkR1^−/−^* mutants (Figure 6D and Figure S9). These results suggest that Sk signaling positively regulates *ILP5* expression. Next, we asked whether downregulation of the *ILP5* affects *Sk* expression. After silencing the *ILP5*, we did not observe any changes in the expression levels of *Sk* and *SkR1* (Figure 6E–G). We also tested the role of *ILP5* in the feeding of *B. dorsalis*. Indeed, we observed that flies with silenced *ILP5* consumed more food than control flies injected with ds*GFP* (Figure 6I). Thus, *ILP5* inhibits feeding. Notably, double fluorescence in situ hybridization results showed that *ILP5* and *SkR1* were co‐localized in the PI region of the brain (Figure 6H). Together with previous findings, we propose that satiated *B. dorsalis* induces Sk release and activates SkR1 by Gr43a sensing up‐regulated fructose in the brain, which in turn promotes the release of ILP5 and inhibits feeding behavior (Figure 7). Additionally, we summarize other studies on Sk regulation of insect feeding behavior at the peripheral level and propose a working model (Figure 7).

## 3 Discussion

In animals, Sk (CCK in vertebrates) signaling to regulate feeding is a complex process that not only modulates the peripheral system sensitivity to alter food acceptability, but also acts as an internal sensor to assess nutrition [12]. In *D. melanogaster* and *Nilaparvata lugens*, Sk acts on the proboscis and proleg tarsi to inhibit sugar attraction, from which it suppresses food consumption [13]. Our recent study has shown that Sk enhances foraging behavior by altering the expression of olfactory receptors in starved *B. dorsalis* [15]. Here, we showed that Sk also acts as an internal nutrient sensor to regulate the consumption of non‐sweet nutritive sugar. Sk neurons receive signal inputs from elevated fructose sensed by Gr43a to inhibit overfeeding, and elevated fructose in the hemolymph may come directly from dietary fructose, or from metabolized other nutritive sugars. Notably, Sk does not modulate the consumption of sweet non‐nutritive sugars, such as sucralose. This suggests that Sk modulation of sweet taste sensitivity needs to be driven by internal nutritional assessment. Thus, the coordination of external taste sensitivity and internal nutrient sensing by Sk to modulate feeding is an interesting question that remains to be investigated.

Which neurotransmitter the *Gr43a* neurons use to communicate with downstream *Sk* neurons remains unclear. Here, we showed that Sk‐mediated feeding mechanisms in which fructose and Gr43a act as internal nutrient cues and nutrient sensors, respectively, in the brain. To clarify how this internal nutrient signaling is conveyed to Sk‐IPC neurons, it is crucial to identify the downstream of the *Gr43a* activation. The *Gr43a* was shown to be expressed in the dorsal lateral peptidergic (DLP) neurons that are partially overlapping with the cells expressing corazonin (Crz), short neuropeptide F (sNPF), and diuretic hormone 31 (Dh31), which innervates the IPC [18, 19, 20]. The sNPF was shown to promote Dilp2 and 5 expression [18]. The link between Gr43a and activation of Sk‐IPC neuron in *Bactocera* needs to be further investigated. In addition, we must note that internal *Gr43a* expressing cells are not only in the brain, but also in the uterus and proventriculus, which may be involved in the process of feeding stimulation [19].

In mammals, it has long been recognized that CCK receptors are abundantly expressed in pancreatic islet cells [21]. In particular, CCK peptides were found to stimulate insulin release [22]. However, no studies have yet demonstrated a direct effect of Sk on insulin cells in insects. Here, we report that Sk‐SkR1 signaling regulates ILP5 expression to inhibit food consumption in *B. dorsalis*. It is also shown that SkR1 is co‐localized with IPCs expressing ILP5. This suggests that Sk inhibits feeding by targeting IPCs, similar to some mammals, including humans [22]. However, the molecular mechanism by which Sk‐SkR1 signaling regulates ILP5 expression remains unknown. In mammals, the transcription factor carbohydrate response element binding protein (ChREBP) regulates fructose‐induced insulin secretion and homeostasis [23, 24]. Mio, which is homologous to ChREBP, regulates feeding by modulating dilps expression in the *Drosophila* brain. *Dilp2*‐Gal4 neuron‐specific knockdown of Mio leads to an increase in dilp3 expression [25]. Therefore, focusing on transcription factors that respond to sugars, especially fructose, and are specifically expressed in the IPCs is an important direction to investigate the molecular mechanisms by which Sk‐SkR1 regulates the expression of ILPs.

## 4 Experimental Section

### 4.1 Insects

The wild‐type (WT) oriental fruit flies used in this study were originally collected from Hainan province of China, and were reared at laboratory conditions of 27.5 ± 0.5°C, 75 ± 5% relative humidity (RH), and a 14:10 h (L:D) photoperiod. Larvae and adults were fed with artificial food as previously described [26].

### 4.2 Quantitative Real‐Time PCR

Brains from flies of different genotypes with different treatments were dissected and immediately stored at −80°C. Total RNA was extracted with Trizol Reagent (Invitrogen) following the manufacturer's protocol. First‐strand cDNA was synthesized using the PrimeScript first‐strand cDNA Synthesis Kit (Takara, Dalian, China) and specific primers designed using the online software, Primer 3 (http://primer3.ut.ee/, Table S1). Quantitative real‐time reverse transcription PCR (qRT‐PCR) was performed using a CFX Connect Real‐Time System (Bio‐Rad, Hercules, CA) with NovoStart SYBR PCR SuperMix kit (Novoprotein, Shanghai, China). Two reference genes, α‐tubulin (GenBank: GU269902) and RPS3 (GenBank: XM_011212815), were selected to normalize the expression levels for the calculation in qBASE [26, 27]. Each experiment comprised three independent biological replicates and two technical replicates.

### 4.3 Immunohistochemistry

Rabbit anti‐Sk antibody was a gift from Dr. Chuan Zhou [28]. Immunohistochemistry was performed as previously described [29]. Brains of 7–10‐day‐old *B. dorsalis* adults were dissected in phosphate‐buffered saline (PBS), fixed overnight in 4% paraformaldehyde (PFA) in PBS at 4°C, and washed (3 × 15 min) in PBST (0.5% Triton X‐100 in PBS). The tissues were blocked in 5% normal goat serum for 20 min at room temperature, then incubated in rabbit anti‐Sk antibody (1: 1000) for 48 h at 4°C. After three washes in PBST (3 × 15 min), the tissues were then incubated in goat anti‐rabbit lgG conjugated to Alexa Fluor 488 (1:1000, Cell Signaling Technology) overnight at 4°C. After washing in PBST again, the tissues were mounted on a slide with 100% glycerol for imaging. Intracellular Sk levels after incubating the brains with sugars were determined as described previously [5]. Male adult brains were dissected in cold sugar free AHL (108 mm NaCl, 8.2 mm MgCl_2_, 4 mm NaHCO_3_,1 mm NaH_2_PO_4_, 2 mm CaCl_2_, 5 mm KCl, 5 mm HEPES), incubated in AHL containing 80 mm trehalose, 80 mm glucose, 80 mm fructose, or 80 mm sucralose (control group) for 30 min, and then fixed in 4% PFA overnight. Finally, the brains were stained with anti‐Sk antibody as described previously. All images were taken with a Leica (STELLARIS 5) confocal microscope and processed with LAS X software. For the quantification of Sk based on fluorescence, we used the same confocal settings for all brains and achieved maximum intensity projection of the Z‐stacks comprising the Sk cell bodies. We then selected the soma regions to measure fluorescence intensity using LAS X software.

### 4.4 Food Intake Assay

Virgin females and males, 3‐day old, were placed each in groups of 25 flies and reared at 27.5°C and 60% humidity, and 9‐day old flies were subjected to perform food‐intake assays. Individual flies were measured by the amount of Bromophenol Blue dye (BPB) in the fly after the food, including 0.2% (BPB), was provided for 1 h for ad libitum feeding, which is long enough time to allow satiation to occur. BPB was measured from decapitated and homogenized bodies in 1 mL PBS buffer with 1% Triton X‐100. Absorbance of the supernatant after centrifugation (13 000 rpm) for 3 min was measured at 614 nm on a xMarker Microplate Spectrophotometer (Bio‐Rad). Images of flies with visually detectable dye in their abdomen were taken by Leica M165C and Leica Microsystems (Germany).

### 4.5 Circulating Fructose Measurements

Fructose was determined by the Seliwanoff's test with modifications [10, 30]. Briefly, adult males were frozen after the feeding procedure, and their heads were homogenized in 150 µL of ultrapure water. The samples were then centrifuged at room temperature at 14 000 rpm for 10 min. 100 µL of supernatant was taken and mixed with 200 µL of 0.05% resorcinol in 6N HCl solution. The mixture was centrifuged at 14 000 rpm for 10 min at room temperature, and the supernatant was incubated at 95°C for 10 min. Finally, the absorbance was measured at 484 nm using a xMarkerTM Microplate Spectrophotometer (Bio‐Rad). Background value (absorption prior to heat treatment) was subtracted to exclude the effect of glucose and trehalose. Three biological replicates were determined for each treatment, and each biological replicate contained 12 male adults.

### 4.6 Genome Editing and Mutant Screening

We identified the guide RNA (gRNA) target site according to the target sequence principle of 5’‐GGN‐18‐NGG‐3’. In vitro synthesis of single guide RNA and embryo microinjection were performed as previously described [29].

Emerged adults (G0) were crossed with wild‐type opposite sex to obtain G1 offspring. Suitable mutant G1 (±) were backcrossed to WT flies for at least five generations, then homozygous mutant flies (‐/‐) were obtained by self‐crossing. Genomic DNA was extracted from individual flies (G0) or a single hind leg (G1 and later generations) by incubating tissue samples in 30 µL InstaGene Matrix (Bio‐Rad, Hercules, CA, USA) at 56°C for 30 min and inactivating the enzyme at 100°C for 10 min. Fragments of 300–500 bp spanning the sgRNA target sites were amplified using the primers listed in Table S1. PCR products were recovered for direct sequencing or cloned into a pGEM‐T Easy Vector (Promega) prior to Sanger sequencing to determine the exact insertion‐deletion types.

### 4.7 Phylogenetic Analysis

Phylogenetic analysis of SkRs was constructed based on amino acid sequences of SkRs and CCK receptors from invertebrates and vertebrates. A phylogenetic tree was constructed using the neighbor‐joining method in MEGA 7. Node support was assessed using a bootstrap procedure based on 1000 replicates. *Homo sapiens* chymotrypsin receptor (SCTR) and vasoactive intestinal polypeptide receptor (VIPR) in goldfish were used as an outgroup.

### 4.8 Identification, Cloning, and Sequence Analysis of SkR1

The full open reading frame (ORF) of BdSkR1 was amplified using high‐fidelity DNA polymerase PrimeSTAR (Takara, Dalian, China). The primers are listed in Table S1. The reaction mixture contained 12.5 µL 2 × PrimerSTAR Max Premix, 1 µL of each forward and reverse gene‐specific primer, 1 µL cDNA template, and 9.5 µL nuclease‐free water, making a final volume of 20 µL. The reaction was heated to 98°C for 2 min, followed by 35 cycles of 98°C for 10 s, 60°C for 15 s, and 72°C for 30 s. Purified PCR products were transferred to the vector pGEM‐T Easy (Promega, Madison, WI) for sequencing (BGI, Beijing, China). Transmembrane helices were predicted using DeepTMHMM (https://services.healthtech.dtu.dk/services/DeepTMHMM‐1.0/) [31]. The sequences were aligned using ClustalW, and a neighbour‐joining tree was constructed in MEGA 11 with 1000 bootstrap replicates [32].

### 4.9 Heterologous Expression and Calcium Assay Functional Assay

Heterologous expression and functional assay were performed as previously described [33]. Briefly, the ORF of the BdSkR1 was inserted into the expression vector pcDNA3.1(+) to construct the plasmid for transient expression. Heterologous expressions of BdSkR1 were made in Chinese hamster ovary (CHO‐WTA11) cells, which were cultured in Dulbecco's modified Eagle medium (DMEM, 11995065, Gibco, Thermo Fisher Scientific, USA) containing 10% Fatal Bovine Serum (FBS, 10091148, Gibco) and 1% penicillin‐streptomycin‐Amphotericin B solution (C0224‐100 mL, Beyotime) with 5% CO2 at 37°C. Cells were collected 30 h later and pre‐incubated with the coelenterazine (Invitrogen) for the functional assay according to the published protocols [34]. Luminescence caused by intracellular calcium mobilization was measured using a TriStar2 LB 942 Multimode Reader (Berthold Technologies, Bad Wildbad, Germany). All experiments were conducted in three biological replicates.

### 4.10 RNA‐Seq Analysis

Total RNA was extracted from the brain tissues of starved WT and *Sk^−/−^* flies using TRIzol reagent (Invitrogen). Three replicates were performed. Library construction and sequencing were performed by Novogene with the Illumina HiSeq2000 platform (Novogene Bioinformatics Technology Co.Ltd, Beijing, China). The raw data (BioProject PRJNA1244087) were analyzed after filtering the low‐quality sequences by fastp with the default parameters [35]. Sequences were aligned to the *B. dorsalis* genome (https://db.cngb.org/search/project/CNP0003192/) using Hisat2 (version 2.2.1) [36, 37]. Samtools (version 1.7) was used to convert sam files to bam files in the process [38]. Finally, to calculate the count, we use featureCounts (version 2.0.1) [39]. The expression level of genes from the RNA sequencing was normalized by the FPKM method. Differential expression analysis was performed using the DESeq2 R package (1.16.1) [40].

### 4.11 RNA Interference Assay

The TranscriptAid T7 High Yield Transcription Kit (K0441, Thermo Scientific, USA) was used for dsRNA synthesis. After the quality of dsRNA was determined on a 1.5% agarose gel, and the final concentration of dsRNA was adjusted to 6.0 µg/µL and delivered to fly at a dose of 1.5 µg by thoracic injection. Control flies were injected with an equal amount of dsGFP. The silencing efficiency of the target gene was assessed by qRT‐PCR 24 h after dsRNA injection. Injections were performed using 7‐day‐old flies, and food intake assays were performed at confirmation of gene disruption (9 days of age). Primers used for the qRT‐PCR assay are listed in Table S1.

### 4.12 In Situ Hybridization

Fluorescence in situ hybridization was performed according to the previous protocol with modifications. Briefly, probes for *SkR1* and *ILP5* were synthesized by the PCR DIG Probe Synthesis Kit version 25 (Roche). Adult brains were dissected in phosphate‐buffered saline (PBS) and fixed overnight in 4% paraformaldehyde (PFA) at 4°C. The brains were then washed (3 × 15 min) with PBST (0.5% Triton X‐100 in PBS), treated with 50 ug/mL Proteinase K for 13 min, and washed again with PBST (3 × 15 min). The brains were re‐fixed in 4% PFA for 2 h, washed in PBST (3 × 15 min), and incubated in hybridization buffer at 48°C for 30 min. Subsequently, the brains were incubated with 200 µL of hybridization buffer (Roche) containing the probes (dilution 1: 20) at 48°C for 24 h. Then the brains were washed with PBST (3 × 15 min), blocked with 5% normal goat serum for 20 min, and incubated with monoclonal anti‐digoxigenin (mouse lgG1 isotype) (1: 1000, Sigma) at 4°C for 48 h. After three washes in PBST (3 × 15 min), the brains were incubated overnight at 4°C in anti‐mouse lgG conjugated to Alexa Fluor 488 (1:1000, Cell Signaling Technology). After washing again with PBST, the brains were mounted on slides with 100% glycerol for imaging. Images were taken with a Leica (STELLARIS 5) confocal microscope and processed with LAS X software.

### 4.13 Statistical Analysis

For qRT‐PCR result analysis, relative expression levels were calculated using qBase based on the expression of the reference genes [27]. Statistical analysis is performed using GraphPad Prism and indicated in each figure legend. Data were first verified for normal distribution using the Shapiro‐Wilk normality test. One‐way ANOVA, followed by Tukey's multiple comparisons, was used for comparisons among multiple groups. Values are reported as means ± standard errors of the mean (SEM).

## Author Contributions

H.F. B.D., Z.R., X.Z., and Q.L. performed the experiments and analyzed data. H.J., J.W., and Y.P. supervised the project and wrote the manuscript. All authors discussed, wrote, and commented on the final version of the manuscript.

## Funding

This research was supported by funding from the National Key R&D Program of China (2022YFC2601000), the National Natural Science Foundation of China (U21A20222, 32472554), 111 Project (B18044), and the China Agriculture Research System of MOF and MARA.

## Conflicts of Interest

The authors declare no conflict of interest.

## Supporting information




**Supporting File**: advs74777‐sup‐0001‐SuppMat.docx.

## Data Availability

The data that support the findings of this study are available in the supplementary material of this article.
