# Supplementary material for: Sulfakinin Signaling Sense Circulating Fructose and Suppresses Food Consumption via Insulin‐Like Peptide in Bactrocera Dorsalis
Source: Adv Sci (Weinh). 2026 Mar 12;13(29):e14748. doi: 10.1002/advs.202514748 (PMC13205718; doi:10.1002/advs.202514748)
Supplement: Supplementary file 1 — Supporting File: advs74777‐sup‐0001‐SuppMat.docx. [file ADVS-13-e14748-s001.docx]

Supplementary Information

**Sulfakinin signaling sense circulating fructose and suppresses food consumption via insulin-like peptide in *Bactrocera dorsalis***

*Hong-Fei Li, Bao Dong, Zheng-Lin Ren, Xue-Guang Zhu, Quan Lei, Yoonseong Park^*^, Jin-Jun Wang^*^, and Hong-Bo Jiang^*^*

Figure S1. Spatio-temporal expression pattern of Sk, SkR1 and SkR2. (A) Expressions of Sk, SkR1 and SkR2 in different development stages of *B. dorsalis*. (B) Expressions of Sk, SkR1 and SkR2 in different external tissues. (C) Expressions of Sk, SkR1 and SkR2 in different internal tissues. Data are shown as the mean ± SEM.

**Figure S2**. Feeding induces Sk mRNA transcription in *B. dorsalis* by *in situ* hybridization. (A) Sk mRNA levels in Sk-MP neurons of starved and refed flies by *in situ* hybridization. Right panel showed representative images of Sk-MP neurons. (B) Sk mRNA levels in Sk-ILP neurons of starved and refed flies by *in situ* hybridization. Right panel showed representative images of Sk-ILP neurons. Sk mRNA levels by *in situ* hybridization were significantly higher after refeeding both in Sk-ILP and Sk-MP neurons. *n* = 3. All data are plotted as mean ± SEM. Unpaired *t* test, **P* < 0.05, ***P* < 0.01.

Figure S3. Linear relation between absorbance and food intake. *n* = 4. Data are plotted as mean ± SEM.

Figure S4. The intensity of Sk immunoreactivity at different time points after feeding. (A) The intensity of immunoreactivity in Sk-MP neurons at 15 minutes, 30 minutes, 1 hour, and 2 hours post-feeding. Right panel showed representative images of Sk-MP neurons. (B) The intensity of immunoreactivity in Sk-ILP neurons at 15 minutes, 30 minutes, 1 hour, and 2 hours post-feeding. Right panel showed representative images of Sk-ILP neurons. *n* = 3 flies. All data are plotted as mean ± SEM. Unpaired *t* test, ns indicates no significant difference, **P* < 0.05, ***P* < 0.01.

Figure S5. Food consumption of WT and *Gr43a^-/-^* flies allowing them to feed *ad libitum* for 24 hours. Knockout of *Gr43a* increased food consumption of flies. *n* = 26 and 24 flies respectively. All data are plotted as mean ± SEM, Unpaired *t*-test; ***P* < 0.01.

Figure S6. Identification and functional characterization of SkR1. (A) Phylogenetic tree of sulfakinin receptors constructed using the neighbor-joining (NJ) method. Numbers shown at branches are percentage of replicate trees in the bootstrap test (1000 replicates). Tree scale indicates an evolutionary distance of 0.1. *Carassius auratus* vasoactive intestinal polypeptide receptor (VIPR) and *Homo sapiens* secretin receptor (SCTR) were used as the outgroup. Accession numbers were shown in Table S4. (B) Deduced amino acid sequences of *B. dorsalis* SkR1. The blue letters indicate the transmembrane domains. (C) Dose-response curves for the Sk1 and Sk2 peptides in the CHO-WTA11 cells, overexpressing the *B. dorsalis* SkR1.

Figure S7. Quantification of anti-Sk signal in Sk-ILP neurons of starved *SkR1^-/-^* and refed *SkR1^-/-^* flies. The intensity of immunoreactivity in Sk-ILP neurons of starved *SkR1^-/-^* flies was the same as that in Sk-ILP neurons of refed *SkR1^-/-^* flies. Right panel showed representative images of Sk-ILP neurons. *n* = 3. All data are plotted as mean ± SEM. Unpaired *t*-test, ns indicates no significant difference.

Figure S8. WEGO (Web Gene Ontology Annotation Plotting) output for Sk regulated genes. The histogram shows the count of genes with GO terms enriched in each category.

Figure S9. The mRNA level of ILP5 in starved and refed *SkR1^-/-^* mutants. The mRNA level of ILP5 in starved *SkR1^-/-^* flies was same as that in refed *SkR1^-/-^* flies. *n* = 4. All data are plotted as mean ± SEM. Unpaired *t*-test, ns indicates no significant difference.

Table S1. Primer sequences used in this study

| Experiments | Gene name | Primer name | Primer sequences (5′ → 3′) |
| --- | --- | --- | --- |
| RT-qPCR | RPS3 | q-RPS3-F | TAAGTTGACCGGAGGTTTGG |
|  |  | q-RPS3-R | TGGATCACCAGAGTGGATCA |
|  | α-tubulin | q-α-tubulin-F | CGCATTCATGGTTGATAACG |
|  |  | q-α-tubulin-R | GGGCACCAAGTTAGTCTGGA |
|  | Sulfakinin | q-Sulfakinin-F | TGGTGGCCTTAACGTTGACT |
|  |  | q-Sulfakinin-R | CCAGAGGCATACCACCAGAT |
|  | SulfakininR1 | q-SulfakininR1-F | TCAAACGAGGCGAAAAATCT |
|  |  | q-SulfakininR1-R | CGTAAATAGCTGGGCCGATA |
|  | SulfakininR2 | q-SulfakininR2-F | TATTGTTGGGGGTCTTCTGC |
|  |  | q-SulfakininR2-R | ATAGCGTTCGCAGGATATGG |
|  | ILP5 | q-ILP5-F | TATGTGCGAAAATGGCTTCA |
|  |  | q-ILP5-R | CCGAGTTTTGGCCACTGTAT |
| RT-qPCR for gene expression in mutants | Sulfakinin | q2-Sulfakinin1-F | GCAGTAACTTGGATGGAATGC |
|  |  | q2-Sulfakinin1-R | GGCCGTAGCCGTATATTGAT |
|  | SulfakininR1 | q2-SulfakininR1-F | GCCTCTCACCTTCGTGTTTC |
|  |  | q2-SulfakininR1-R | GCGAGATTCAGCAGGAAGAC |
| Probe synthesis | ILP5 | ISH-ILP5-F | CTATGATCTCGAGTGCGCTG |
|  |  | ISH-ILP5-R | GTAAGCCTATTTGCAGTAGG |
|  | SulfakininR1 | FISH-SKR1-F | TGCCATTTTCGGGAATCTAC |
|  |  | FISH-SKR1-R | CGTTCATACGCAATGGTGTC |
| dsRNA synthesis | dsGFP | dsGFP-F | TaatacgactcactatagggTGAGCAAGGGCGAGGAGCTG |
|  |  | dsGFP-R | TaatacgactcactatagggTCGATGCGGTTCACCAG |
|  | dsILP5 | dsILP5-F | taatacgactcactatagggTGCGCTGAAAATTGTTACCC |
|  |  | dsILP5-R | taatacgactcactatagggATCAAGCCGAAAGGTGACAG |
| Mutant screening | Sulfakinin | Sk-F | TTGAGCATTCAAAACGAAGC |
|  |  | Sk-R | AAACATTTTCGGGCCGTAG |
|  | SulfakininR1 | SkR1-F | GCGAACTGTTGAACGAGGAT |
|  |  | SkR1-R | AGCAGTGTGCCGACTAAGGT |

Table S2. Statistical table of sequencing data

| Sample | Raw reads | Clean reads | Clean bases | Error rate (%) | Q20 (%) | Q30 (%) | GC content (%) |
| --- | --- | --- | --- | --- | --- | --- | --- |
| WT-1 | 40,028,604 | 39,218,630 | 5.88G | 0.03 | 97.91 | 93.75 | 38.94 |
| WT-2 | 55,415,452 | 54518,082 | 8.18G | 0.03 | 97.31 | 92.53 | 38.60 |
| WT-3 | 50,142,284 | 49,227,274 | 7.38G | 0.03 | 97.39 | 92.74 | 38.64 |
| *Sk^-/-^*-1 | 41,798,588 | 41,096,488 | 6.16G | 0.03 | 97.88 | 93.65 | 38.85 |
| *Sk^-/-^*-2 | 48,998,374 | 48,747,392 | 7.31G | 0.02 | 98.13 | 94.27 | 38.29 |
| *Sk^-/-^*-3 | 47,503,786 | 46,607,082 | 6.99G | 0.03 | 98.04 | 94.08 | 38.30 |

**Table S3.** The result of sequencing data and genome sequence comparison

| Sample | Total Reads | Mapped Reads | Unique Mapped Reads | Multiple Map Reads |
| --- | --- | --- | --- | --- |
| WT-1 | 39,218,630 | 33,562,118 (85.58%) | 31,323,323 (79.87%) | 2,238,795 (5.71%) |
| WT-2 | 54518,082 | 46,203,963 (84.75%) | 43,114,120 (79.08%) | 3,089,843 (5.67%) |
| WT-3 | 49,227,274 | 41,711,001 (84.73%) | 38,897,504 (79.02%) | 2,813,497 (5.72%) |
| *Sk^-/-^*-1 | 41,096,488 | 34,863,030 (84.83%) | 32,778,543 (79.76%) | 2,084,487 (5.07%) |
| *Sk^-/-^*-2 | 48,747,392 | 41,587,654 (85.31%) | 38,853,428 (79.7%) | 2,734,226 (5.61%) |
| *Sk^-/-^*-3 | 46,607,082 | 39,647,709 (85.07%) | 37,375,175 (80.19%) | 2,272,534 (4.88%) |

Table S4. Protein used in the receptor alignment and phylogenetic analysis.

| Species | Protein | Accession number | Species | Protein | Accession number |
| --- | --- | --- | --- | --- | --- |
| *Drosophila melanogaster* | SKR1 | NP_001097023.1 | *Harpegnathos saltator* | SKR | EFN85362.1 |
|  | SKR2 | NP_001097021.1 | *Periplaneta americana* | SKR | AAX56942.1 |
| *Tribolium castaneum* | SKR1 | XP_975226.2 | *Macaca mulatta* | CCK1R | XP_001084186.1 |
|  | SKR2 | XP_972750.1 |  | CCK2R | XP_001102094.1 |
| *Zophobas morio* | SKR1 | XP_063923366.1 | *Homo sapiens* | CCK1R | NP_000721.1 |
|  | SKR2 | XP_063903995.1 |  | CCK2R | NP_795344.1 |
| *Rhodnius prolixus* | SKR1 | QEI59506.1 | *Danio rerio* | CCK1R | XP_697493.2 |
|  | SKR2 | QEI59507.1 |  | CCK2R | CAQ14219.1 |
| *Bactrocera dorsalis* | SKR1 | XP_049310317.1 | *Gallus gallus* | CCK1R | NP_001074970.1 |
|  | SKR2 | XP_049311206.1 |  | CCK2R | NP_001001742.1 |
| *Bombyx mori* | SKR | NP_001127744.1 | *Carassius auratus* | VIPR | AAB05459.1 |
| *Nilaparvata lugens* | SKR | BAO01059.1 | *Homo sapiens* | SCTR | AAA64949.1 |
| *Aedes aegypti* | SKR | XP_021694216.1 |  |  |  |
